# Postoperative outcomes in patients operated for extra- and intracapsular hip fractures – a secondary analysis of two randomized controlled trials

**DOI:** 10.1186/s12891-025-08404-6

**Published:** 2025-02-22

**Authors:** Shams Dakhil, Ane Djuv, Ingvild Saltvedt, Torgeir Bruun Wyller, Frede Frihagen, Lars Gunnar Johnsen, Kristin Taraldsen, Jorunn L. Helbostad, Leiv Otto Watne, Aksel Paulsen

**Affiliations:** 1 Institute of Clinical Medicine, University of Oslo, Campus Ahus, Oslo, Norway; 2 Oslo Delirium Research Group, Department of Geriatric Medicine, Oslo University Hospital, Oslo, Norway; 3 Department of Orthopaedic Surgery, Stavanger University Hospital, Stavanger, Norway; 4 Department of Clinical Medicine, Faculty of Medicine, University of Bergen, Bergen, Norway; 5 Department of Geriatric Medicine, St. Olav University Hospital, Trondheim, Norway; 6 Department of Neuromedicine and Movement Science, Norwegian University of Science and Technology (NTNU), Trondheim, Norway; 7 Department of Orthopaedic Surgery, Østfold Hospital Trust, Grålum, Norway; 8 Orthopedic Trauma Unit, Department of Orthopedic Surgery, St. Olav University Hospital, Trondheim, Norway; 9 Department of Rehabilitation Science and Health Technology, OsloMet – Oslo Metropolitan University, Oslo, Norway; 10 Department of Geriatric Medicine, Akershus University Hospital, Lørenskog, Norway; 11 Department of Public Health, Faculty of Health Sciences, University of Stavanger, Stavanger, Norway

**Keywords:** Hip fracture, Proximal femoral hip fracture, Extracapsular hip fracture, Intracapsular hip fracture, Mortality

## Abstract

**Background:**

Hip fractures are among the most common and serious injuries in older adults. There has been a perception that extracapsular hip fractures have worse outcome than intracapsular hip fractures. We aimed to examine postoperative outcomes in patients operated for extra- and intracapsular hip fractures.

**Methods:**

This is a secondary analysis of data from two randomized controlled trials evaluating the effect of orthogeriatric care. Bivariate analyses were conducted, comparing patients with extracapsular fracture to patients with intracapsular fracture. Mortality, length of hospital stay (LOS), new nursing home admissions, operative data and measures of functional and cognitive performance were assessed as endpoints.

**Results:**

The primary analysis included 711 patients; 283 patients had an extracapsular fracture and 428 an intracapsular fracture. At four months follow-up, the intracapsular fracture group had significantly better Short Physical Performance Battery (SPPB) (5.0 vs. 4.0, *p* = 0.007), personal Activities of Daily Living (p-ADL) (17.0 vs. 16.0, *p* = 0.007) and instrumental ADL (i-ADL) (32.5 vs. 28.0, *p* = 0.049). There were no statistically significant differences between the groups at 12 months.

**Conclusions:**

Patients with an extracapsular fracture had worse mobility and ADL levels four months postoperatively, but there were no clinically relevant differences at 12 months postoperatively.

**Supplementary Information:**

The online version contains supplementary material available at 10.1186/s12891-025-08404-6.

## Background

Hip fractures are among the most common fractures in adults above 50 years of age [1, 2], and are rightfully one of the most feared fractures – due to a risk of loss of independence, complications and death [3, 4]. With increase in life expectancy, the number of these fractures is expected to increase [5]. The mean age of hip fracture patients in Norway is 82 years; 83 years for women and 81 years for men [6]. The comorbidity in this patient group is high, and dementia, sarcopenia and osteoporosis are common [7, 8, 9, 10, 11]. The one-year mortality rate after a hip fracture is 20–25%. Patients with an extracapsular hip fracture have been reported to have more pain and higher mortality than those with intracapsular fractures [12, 13]. In Norway, extracapsular fractures of the femur represent about 40% of the hip fractures [14].

With few exceptions, hip fractures in adults are treated surgically in Western countries. Arthroplasty is the main treatment for intracapsular fractures, with internal fixation being used for special indications, such as young patients or undisplaced or minimally displaced fractures [15, 16, 17]. For extracapsular fractures, the procedure of choice is closed, or occasionally open, reduction followed by either an intramedullary nail or a sliding hip screw [18, 19, 20, 21]. Surgical complications are usually reported to be below 5% after arthroplasty, above 5% after internal fixation, and above 10% in the case of internal fixation of intracapsular fractures and unstable extracapsular fractures [15, 18, 19]. Even in the absence of a defined surgical complication, shortening, rotational deformities and prolonged pain are among the problems with internal fixation [22, 23, 24, 25].

The hip fracture population is heterogeneous regarding demographics, rehabilitation, mortality and postoperative recovery [26, 27]. Moreover, the evidence suggests that the two hip fracture types (extracapsular and intracapsular) should be addressed and treated independently [28]. Therefore, in addition to different needs in operation method, there is some documentation that patients with an extracapsular fracture are older and frailer, experience more blood loss before surgery [29, 30], are more dependent at the time of fracture, and require more resources and time in rehabilitation [28, 31, 32]. In addition, patients with an extracapsular hip fracture are reported to have higher mortality, less ability to bear weight on the affected leg, impaired mobility and lower odds of regaining their independence in personal Activities of Daily Living (p-ADL) at discharge, and a higher rate of institutionalization [33, 34, 35].

Mortality has been shown to be similar in intra- and extracapsular hip fractures patients, as has average LOS, but with a multimodal LOS distribution for the extracapsular hip fractures patients [36]. Intra- and extracapsular hip fractures patients are two distinct subpopulations [28], who require different rehabilitation to achieve a similar functional gain [32]. Despite large studies on LOS for these patient groups, nationally representative dataset from low attrition, trial-based cohorts with high follow-up and extensive functional- and cognitive performance measures are warranted. By combining two trial datasets we can assess variations in outcome and discuss differences in rehabilitation.

Our aim was to examine postoperative outcomes in patients operated for extra- and intracapsular hip fractures in terms of mortality, new nursing home admissions, length of hospital stay (LOS), and cognitive and functional outcomes. We compared extracapsular fractures to intracapsular fractures in a hip fracture population in Norway.

## Methods

### Design

This study is based on data from two Norwegian randomized controlled clinical trials aiming to investigate the effect of orthogeriatric care, randomizing hip fracture patients to usual care in an orthopedic ward or to intervention in a geriatric ward [12, 13]. The Oslo Orthogeriatric Trial included 329 patients between 2009 and 2012 and The Trondheim Hip Fracture Trial included 397 patients between 2008 and 2010. Both studies included only hip fractures due to low-energy trauma. The Oslo study included all patients, at all ages, whilst the Trondheim study included home-dwelling patients above 70 years. These studies were planned in concert with similar design for future pooling of data [37, 38]. Patients were assessed at baseline, four and 12 months after surgery. In the Oslo Orthogeriatric Trial, there was no effect of the intervention on the primary outcome (cognitive function), but there was an effect on mobility four months after surgery for home-dwelling patients [13]. The Trondheim Hip Fracture Trial found better mobility and instrumental Activities of Daily Living (i-ADL) in the intervention group four months after surgery, as well as finding the intervention to be beneficial for most secondary outcomes and being cost-effective up to one year after surgery [12].

### Sample and setting

Data from the Oslo Orthogeriatric Trial and the Trondheim Hip Fracture Trial were pooled, resulting in a larger, more heterogeneous and representative database for hip fracture patients [39]. For the primary analysis of postoperative outcomes, comparing extracapsular hip fractures with intracapsular hip fractures, we included all patients in the database, at all ages, operated with HA or internal fixation, and excluded patients that were not operated (*n* = 7), operated with a total hip arthroplasty (*n* = 7) or an excision arthroplasty (*n* = 1), or where information on fracture type and operation method was missing (*n* = 1).

In the sub-group analyses, we included patients ≥ 70 years old and sub-groups were further based on operation method. Due to change in the treatment of intracapsular hip fractures after a study published by Dolatowski et al. in 2016, intracapsular fractures fixated with osteosynthesis were excluded in the sub-group analyses (*n* = 124) [40]. At the four- and 12-months assessments, patients were assessed at the outpatient clinics at the hospital. For participants unable to visit the hospital, the assessors offered home visits.

### Descriptive measures

Descriptive measures were demographic data (age (years) and sex (male/female)), Body Mass Index (BMI) and place of residence. The American Society of Anesthesiologists (ASA) score [41] and the Charlson Comorbidity Index (CCI) [42] were used to assess comorbidity at baseline. In addition, measures of functional (i-ADL and personal ADL (p-ADL)), physical (hand grip strength) and cognitive (The Clinical Dementia Rating Scale (CDR)) performance were assessed at baseline, see section below.

### Outcomes

Hospital outcomes included length of hospital stay (LOS) in days and operative data including type of fracture (extracapsular vs. intracapsular), waiting time to operation (hours), duration of operation (measured as the time from start to stop of anesthesia), type of anesthesia (local/regional vs. general) and operation method (HA vs. internal fixation).

Outcomes assessed at four- and 12-months follow up were place of residence, mortality rate and measures of functional, physical and cognitive performance, as well as depressive symptoms.

Place of residence was categorized into whether or not the patients were living in a nursing home at follow-up. Functional outcomes included i-ADL measured by the Nottingham Extended ADL (NEADL) [43] a 22-item questionnaire with scores ranging from 0 to 66 [43], and p-ADL measured by the Barthel ADL Index (BADL) [44] a 10-item questionnaire (range from 0 to 20) [44]. Higher score represents better function. For both i-ADL and p-ADL, the baseline value was obtained by proxy interview, and represents the patient’s pre-fracture function. The values at follow-up were obtained either by proxy-interview or interview of the patient.

The Short Physical Performance Battery (SPPB) [45], gait speed (derived from the SPPB) and hand grip strength were used to measure physical function. SPPB and gait speed was assessed at the 4- and 12 months follow-up, and hand grip strength was assessed postoperative during the hospital stay and at 4 and 12 months follow-up. The SPPB has three domains (balance, sit to stand and gait speed) for a maximum score of 12, in which higher score represents better mobility [45]. Hand grip strength was measured using hand dynamometry (JAMAR, Germany).

CDR [46] at baseline (reflecting pre-fracture cognitive function) and at 4 and 12 months postoperatively, as well as the Mini Mental State Evaluation (MMSE) [47] at 4 and 12 months postoperatively were used to assess cognitive function. The CDR consists of six domains, each scored 0–3, and is summarized for a total score of maximum 18, which is called the sum of boxes score [46]. A higher score indicates more severe dementia. MMSE is a screening test for cognitive impairment (range 0–30), in which a higher score indicates better cognitive function [47].

Depressive symptoms were assessed with the Cornell Scale of Depression in Dementia (CSDD) [48, 49] in Oslo, and the Geriatric Depression Scale 15 (GDS-15) [50] in Trondheim, higher score on each of the scales indicates more severe depressive symptoms. For this study, we merged the two scales by using a cut-off for each scale (CSDD cut-off ≥ 8 [48, 49], GDS-15 cut-off ≥ 6 [51]) and dichotomized the outcome into yes or no for depressive symptoms.

### Statistical methods

We compared postoperative outcomes between extracapsular hip fracture patients and intracapsular hip fracture patients. In a subgroup analysis of patients above 70 years, we assessed outcomes between extracapsular hip fracture patients and intracapsular hip fracture patients operated with HA. and also compared intracapsular hip fracture patients operated with HA to those with internal fixation.

Medians and interquartile range (IQR), as well as rates and percentages are reported. All outcomes were analyzed by the Mann-Whitney U-test and the Chi-square test, depending on data distribution. Time based outcomes (Length of stay and duration of surgery) where in addition analyzed with Kaplan-Meier survival analysis and Log-Rank tests. A two-sided p-value below 0.05 was considered to indicate statistical significance. Imputation was performed for missing items within the NEADL and BADL scales. This was done by imputing the mean score for the remaining items that were answered, if at least 80% of the items on the scale were answered. A sensitivity analysis was performed with patients admitted from a nursing home excluded. All data analyses were performed using IBM Statistics version 28.0.

## Results

### Description of the study population

In total, 711 patients were included, of whom 283 had an extracapsular and 428 an intracapsular fracture (299 operated with HA and 128 with internal fixation and 1 with this information missing). Median age was 85.0 years (range 46–99) for the extracapsular fracture group, and 84.0 years (range 57–101) for the intracapsular fracture group. The majority of patients were female (74.9% in the extracapsular fracture group and 74.3% in the intracapsular fracture group), and 15.9% vs. 13.1% were living in a nursing home at admission in the extracapsular and intracapsular fracture group, respectively (Table 1). The vast majority underwent surgery in local/regional anesthesia (> 95% in both groups).

**Table 1:** Patient characteristics

	Extracapsular fracture *N* = 283	Intracapsular fracture *N* = 428	*p*-value
Age (years), median (range)	85.0 (46 to 99)	84.0 (57 to 101)	0.43^1^
Gender, female, *n* (%)	212 (74.9)	318 (74.3)	0.93^2^
Living in nursing home^a^, *n* (%)	45 (15.9)	56 (13.1)	0.32^2^
BMI^b^, median (IQR)	23.8 (21.4 to 27)	23.5 (20.7 to 26.4)	0.32^1^
ASA score^c^, median (IQR)	3.0 (2 to 3)	3.0 (2 to 3)	0.28^1^
CCI, median (IQR)	1.0 (0 to 3)	2.0 (0 to 3)	0.17^1^
Grip strength^d^ (kg), median (IQR)	18.0 (14 to 25)	20.0 (14 to 26.5)	0.25^1^
p-ADL (BADL)^e^, median (IQR)	18.0 (15.3 to 20)	19.0 (16 to 20)	0.05^1^
i-ADL (NEADL)^f^, median (IQR)	37.0 (19 to 54)	40.5 (20 to 57)	0.30^1^
CDR^g^, sum of boxes, median (IQR)	2.0 (0 to 7)	1.5 (0 to 7)	0.76^1^
Type of anesthesia, n (%)^h^			0.45^2^
- General	14 (5)	16 (4)	
- Local/regional	260 (95)	403 (96)	

*IQR* Inter quartile range. *BMI* Body Mass Index. *ASA* American Society of Anesthesiologists. *CCI* Charlson Comorbidity Index. *P-ADL* Personal Activities of Daily Living. *BADL* Barthel Activities of Daily Living Index. *I-ADL* Instrumental Activities of Daily Living. *NEADL* Nottingham Extended Activities of Daily Living. *CDR* Clinical Dementia Rating Scale

^a^ Patients admitted from nursing homes were excluded in Trondheim

^b^ Missing in 118 patients with an extracapsular fracture, and 184 patients with an intracapsular fracture

^c^ Missing in 10 patients with an extracapsular fracture, and 13 patients with an intracapsular fracture

^d^ Missing in 29 patients with an extracapsular fracture, and 51 patients with an intracapsular fracture

^e^ Missing in 4 patients with an extracapsular fracture, and 9 patients with an intracapsular fracture

^f^ Missing in 6 patients with an extracapsular fracture, and 12 patients with and intracapsular fracture

^g^ Missing in 19 patients with an extracapsular fracture, and 29 patients with and intracapsular fracture

^h^ Missing in 9 patients with an extracapsular fracture, and 9 patients with and intracapsular fracture

^1^ Mann-Whitney U Test

^2^ Chi-Square Test

670 patients were included in the sub-group analysis limited to patients ≥ 70 years; 268 with an extracapsular and 278 with an intracapsular fracture operated with HA and 124 with an intracapsular fracture operated with internal fixation. Patient characteristics for the sub-group analyses are listed in Table 2, and patient characteristics for sensitivity analysis in Supplementary Table 1.

**Table 2:** Patient characteristics for the sub-group analysis, excluding patients < 70 years old

	Extracapsular fracture *N* = 268	Intracapsular fracture (hemiarthroplasty) *N* = 278	Intracapsular fracture (internal fixation) *N* = 124	*P*-value (A)	*P*-value (B)
Age (years), median (range)	85.0 (70 to 99)	85.0 (70 to 101)	84.5 (70 to 99)	0.53^1^	0.79^1^
Gender, female, n (%)	206 (76.9)	214 (77.0)	87 (70.2)	1.0^2^	0.17^2^
Living in a nursing home^a^, n (%)	44.0 (16.4)	35 (12.6)	19 (15.3)	0.23^2^	0.53^2^
BMI^b^, median (IQR)	23.8 (21.4 to 27.0)	24.4 (21.8 to 26.6)	21.7 (19.6 to 25.5)	0.88^1^	< 0.001^1^
ASA score^c^, median (IQR)	3.0 (2 to 3)	3.0 (2 to 3)	3.0 (2 to 3)	0.29^1^	0.64^1^
CCI, median (IQR)	1.0 (0 to 3)	2.0 (0 to 3)	2.0 (1 to 4)	0.62^1^	0.008^1^
Grip strength^d^ (kg), median (IQR)	18.0 (13.3 to 24)	18.0 (14 to 24)	20.0 (13.3 to 27.5)	0.31^1^	0.80^1^
p-ADL (BADL)^e^, median (IQR)	18.0 (15 to 20)	19.0 (16 to 20)	19.0 (15.8 to 20)	0.08^1^	0.93^1^
i-ADL (NEADL)^f^, median (IQR)	37.0 (17.8 to 54)	39.0 (19.5 to 56)	40.5 (19.8 to 57)	0.64^1^	0.50^1^
CDR^g^, sum of boxes, median (IQR)	2.0 (0 to 7)	2.0 (0 to 7)	1 (0 to 7)	0.99^1^	0.59^1^

*IQR* Inter quartile range. *BMI* Body Mass Index. *ASA* American Society of Anesthesiologists. *CCI* Charlson Comorbidity Index. *p-ADL* Personal Activities of Daily Living. *BADL* Barthel Activities of Daily Living Index. *i-ADL* Instrumental Activities of Daily Living. *NEADL* Nottingham Extended Activities of Daily Living. *CDR* Clinical Dementia Rating Scale

^a^ All patients admitted from a nursing home were excluded in Trondheim

^b^ Missing in 114 patients with an extracapsular fracture, 117 patients with an intracapsular fracture operated with hemiarthroplasty, and 61 patients with an intracapsular fracture operated with internal fixation

^c^ Missing in 8 patients with an extracapsular fracture, 5 patients with an intracapsular fracture operated with hemiarthroplasty, and 6 patients with an intracapsular fracture operated with internal fixation

^d^ Missing in 28 patients with an extracapsular fracture, 33 patients with an intracapsular fracture operated with hemiarthroplasty, and 16 patients with an intracapsular fracture operated with internal fixation

^e^ Missing in 4 patients with an extracapsular fracture, 6 patients with an intracapsular fracture operated with hemiarthroplasty, and 2 patients with an intracapsular fracture operated with internal fixation

^f^ Missing in 6 patients with an extracapsular fracture, 9 patients with an intracapsular fracture operated with hemiarthroplasty, and 2 patients with an intracapsular fracture operated with internal fixation

^g^ Missing in 18 patients with an extracapsular fracture, 16 patients with an intracapsular fracture operated with hemiarthroplasty, and 9 patients with an intracapsular fracture operated with internal fixation

^1^ Mann-Whitney U Test

^2^ Chi-Square Test

### Extracapsular vs. intracapsular hip fracture

There was no difference between the groups regarding waiting time for surgery or LOS, see Table 3 and supplementary Table 2. At four-month follow-up, the intracapsular fracture group had significantly better SPPB (5.0 vs. 4.0, *p* = 0.007), p-ADL (17.0 vs. 16.0, *p* = 0.007) and i-ADL (32.5 vs. 28.0, *p* = 0.049) scores (Table 3). This did not persist at 12-months follow-up, where there was no difference between the groups in SPPB, gait speed, BADL or NEADL (Table 3). There were no other differences between the groups at four-months and 12-months follow-up (Table 3). This difference at the early rehabilitation stage might be because hip arthroplasty allows the patients to early full mobilization and weight bearing without crutches or other walking aids [52]. This points towards a more rapid rehabilitation in the intracapsular group and this difference recovery profiles suggests the early and intensive rehabilitation is particularly important in the extracapsular group.

**Table 3:** Outcomes at hospital, 4- and 12-months follow-up

	Extracapsular fracture	Intracapsular fracture	Group differences (*p*-value)
	**Hospital outcomes**		
**N**	**283**	**428**	
Hours to surgery^a^, median (IQR)	23.3 (15 to 39)	24.0 (16 to 40.7)	0.31^1^
Duration of operation (min)^b^, median (IQR)	144.5 (124 to 173.8)	165 (123 to 192.3)	0.001^1^
Died during hospital stay, n (%)	3 (1.1)	9 (2.1)	0.3^2^
Type of anesthesia^c^, local/regional, n (%)	260 (91.9)	403 (94.2)	0.41^2^
Type of operation^d^ Hemiarthroplasty, *n* (%) Internal fixation, *n* (%)	4 (1.4) 279 (98.6)	299 (69.9) 128 (29.9)	< 0.001
Length of hospital stay in days, median (IQR)	10.0 (7 to 14)	10.0 (7 to 13)	0.16^1^
	**4-month follow-up**		
**N**	**228**	**352**	
Living in a nursing home^e^, n (%)	73 (25.8)	97 (22.7)	0.32^2^
Died before 4-month follow-up, n (%)	35 (12.4)	48 (11.2)	0.64^2^
Grip strength^f^ (kg), median (IQR)	18.0 (13 to 24)	20.0 (14 to 25)	0.06^1^
SPPB^g^, median (IQR)	4.0 (1 to 6)	5.0 (2 to 7)	0.007^1^
Gait speed^h^ (m/s), median (IQR)	0.55 (0.4 to 0.7)	0.58 (0.4 to 0.7)	0.09^1^
MMSE^i^, median (IQR)	23.8 (19 to 28)	24.0 (19.5 to 28)	0.60^1^
p-ADL (BADL)^j^, median (IQR)	16.0 (12 to 19)	17.0 (14 to 20)	0.007^1^
i-ADL (NEADL)^k^, median (IQR)	28.0 (8 to 45)	32.5 (11 to 49.8)	0.049^1^
CDR^l^, sum of boxes, median (IQR)	2.0 (0 to 8.3)	1.8 (0 to 7)	0.66^1^
Depression^m^ (%)	119 (42.0)	182 (42.5)	0.82^2^
	**12-month follow-up**		
**N**	**195**	**304**	
Living in a nursing home^e^, n (%)	60 (21.2)	92 (21.5)	0.96^2^
Died before 12-month follow-up, n (%)	62 (21.9)	83 (19.4)	0.42^2^
Grip strength^f^ (kg), median (IQR)	18.0 (12.5 to 24)	18.0 (14 to 24.8)	0.42^1^
SPPB^g^, median (IQR)	4.0 (1 to 6.3)	4.0 (2 to 8)	0.20^1^
Gait speed^h^ (m/s), median (IQR)	0.56 (0.4 to 0.8)	0.58 (0.4 to 0.8)	0.23^1^
MMSE^i^, median (IQR)	25.0 (18 to 28)	24.0 (19 to 27)	0.37^1^
p-ADL (BADL)^j^, median (IQR)	17.0 (12.1 to 19)	17.5 (13.8 to 20.0)	0.36^1^
i-ADL (NEADL)^k^, median (IQR)	28.0 (10 to 48)	33.0 (11 to 51.8)	0.26^1^
CDR^l^, sum of boxes, median (IQR)	1.5 (0 to 9)	2.0 (0 to 10)	0.98^1^
Depression^m^ (%)	103 (36.4)	177 (41.4)	0.27^2^

*IQR* Inter quartile range. *SPPB* Short Physical Performance Battery. *MMSE* Mini Mental State Evaluation. *p-ADL* Personal Activities of Daily Living. *BADL* Barthel Activities of Daily Living Index. *i-ADL* Instrumental Activities of Daily Living. *NEADL* Nottingham Extended Activities of Daily Living. *CDR* Clinical Dementia Rating Scale

^a^ Measured as hours from admission to start of anesthesia. Missing in 2 patients with an intracapsular fracture

^b^ Measured as duration of anesthesia. Missing in 11 patients with an extracapsular fracture, and 14 patients with an intracapsular fracture

^c^ Missing in 9 patients with an extracapsular fracture, and 9 patients with an intracapsular fracture

^d^ Missing in 1 patient with an intracapsular fracture

^e^ Missing in 1 patient with an extracapsular fracture, and 1 patient with an intracapsular fracture at 4-months follow-up. Missing in 1 patient with an intracapsular fracture at 12-months follow-up

^f^ Missing in 36 patients with an extracapsular fracture, and 56 patients with an intracapsular fracture at 4-months follow-up. Missing in 38 patients with an extracapsular fracture and 60 patients with an intracapsular fracture at 12-months follow-up

^g^ Missing in 9 patients with an extracapsular fracture, and 19 patients with an intracapsular fracture at 4-months follow-up. Missing in 17 patients with an extracapsular fracture and 16 patients with an intracapsular fracture at 12-months follow-up

^h^ Missing in 42 patients with an extracapsular fracture, and 55 patients with an intracapsular fracture at 4-months follow-up. Missing in 38 patients with an extracapsular fracture and 52 patients with an intracapsular fracture at 12-months follow-up

^i^ Missing in 15 patients with an extracapsular fracture, and 35 patients with an intracapsular fracture at 4-months follow-up. Missing in 17 patients with an extracapsular fracture and 21 patients with an intracapsular fracture at 12-months follow-up

^j^ Missing in 6 patients with an extracapsular fracture, and 10 patients with an intracapsular fracture at 4-months follow-up. Missing in 7 patients with an extracapsular fracture and 12 patients with an intracapsular fracture at 12-months follow-up

^k^ Missing in 13 patients with an extracapsular fracture, and 16 patients with an intracapsular fracture at 4-months follow-up. Missing in 5 patients with an extracapsular fracture and 12 patients with an intracapsular fracture at 12-months follow-up

^l^ Missing in 15 patients with an extracapsular fracture, and 32 patients with an intracapsular fracture at 4-months follow-up. Missing in 13 patients with an extracapsular fracture and 13 patients with an intracapsular fracture at 12-months follow-up

^m^ Missing in 11 patients with an extracapsular fracture, and 26 patients with an intracapsular fracture at 4-months follow-up. Missing in 17 patients with an extracapsular fracture and 23 patients with an intracapsular fracture at 12-months follow-up

^1^ Mann-Whitney U Test

^2^ Chi-Square Test

### Extracapsular hip fracture vs. intracapsular hip fracture operated with hemiarthroplasty

There was no difference in waiting time for surgery or LOS during hospital stay between the groups, see Table 4. Patients with an intracapsular fracture operated with a HA had longer duration of surgery, measured as the time from start to end of anesthesia (179 min vs. 144 min, *p* < 0.001) (Table 4 and supplementary Table 2).

**Table 4:** Sub-group analysis excluding patients < 70 years old. Outcomes during hospital stay, 4- and 12-months follow-up

	Extracapsular fracture	Intracapsular fracture (hemiarthroplasty)	Intracapsular fracture (internal fixation)	*p*-value (A)	*p*-value (B)
	**Hospital outcomes**				
**N**	**268**	**278**	**124**		
Hours to surgery^a^, median (IQR)	23.0 (15 to 38.4)	25.0 (17 to 40)	23.0 (14.3 to 41)	0.14^1^	0.28^1^
Duration of operation (min)^b^, median (IQR)	144 (124 to 174)	179 (156.3 to 200)	107 (88.5 to 141)	< 0.001^1^	< 0.001^1^
Type of anesthesia^c^, local/regional (%)	251 (93.7)	263 (94.6)	116 (93.5)	0.75^2^	0.96^2^
Type of operation Hemiarthroplasty, *n* (%) Internal fixation, *n* (%)	3 (1.1) 265 (98.1)	278 (100) -	- 124 (100)	-	-
Length of hospital stay in days, median (IQR)	10.0 (7 to 14)	11 (8 to 14)	8 (6 to 12)	0.86^1^	< 0.001^1^
Died during hospital stay, n (%)	3 (1.1)	9 (3.2)	0 (0)	0.09^2^	0.043^2^
	**4-month follow-up**				
**N**	**215**	**222**	**110**		
Living in a nursing home^d^, n (%)	72 (26.9)	61 (21.9)	34 (27.4)	0.21^2^	0.45^2^
Died before 4-month follow-up, *n* (%)	34 (12.7)	34 (12.2)	13 (10.5)	0.87^2^	0.62^2^
Grip strength^e^ (kg), median (IQR)	17.0 (12 to 22.5)	18.0 (14 to 24)	20.0 (14 to 24)	0.08^1^	0.86^1^
SPPB^f^, median (IQR)	3.0 (1 to 6)	4.0 (2 to 7)	4.5 (2 to 8)	0.014^1^	0.72^1^
Gait speed^g^ (m/s), median (IQR)	0.53 (0.4 to 0.7)	0.56 (0.4 to 0.7)	0.6 (0.4 to 0.8)	0.23^1^	0.25^1^
MMSE^h^, median (IQR)	23.0 (19 to 28)	24.0 (19 to 28)	24.0 (19 to 28)	0.56^1^	0.82^1^
p-ADL (BADL)^i^, median (IQR)	16.0 (11 to 19)	17.0 (14 to 19)	18.0 (12 to 20)	0.014^1^	0.92^1^
i-ADL (NEADL)^j^, median (IQR)	27.0 (8 to 44)	29.0 (11 to 47)	30.0 (9 to 49.3)	0.09^1^	0.99^1^
CDR^k^, sum of boxes, median (IQR)	2.0 (0 to 9)	2.0 (0 to 8)	2.0 (0 to 8)	0.77^1^	0.70^1^
Depressive symptoms^l^, n (%)	119 (44.4)	119 (42.8)	61 (49.2)	1.0^2^	0.77^2^
	**12-month follow-up**				
**N**	**184**	**195**	**92**		
Living in a nursing home^d^, n (%)	59 (22.0)	61 (21.9)	30 (24.2)	0.96^1^	0.72^1^
Died before 12-month follow-up, n (%)	60 (22.4)	54 (19.4)	25 (20.2)	0.39^2^	0.86^2^
Grip strength^e^ (kg), median (IQR)	17.0 (12 to 23.5)	18.0 (13 to 24)	18.0 (14 to 24)	0.45^1^	0.89^1^
SPPB^f^, median (IQR)	4.0 (1 to 6)	4.0 (2 to 7)	4.0 (1 to 8)	0.47^1^	0.62^1^
Gait speed^g^ (m/s), median (IQR)	0.55 (0.4 to 0.8)	0.56 (0.4 to 0.7)	0.64 (0.5 to 0.9)	0.98^1^	0.009^1^
MMSE^h^, median (IQR)	25.0 (18 to 28)	24.0 (18 to 27)	24.0 (19.7 to 27.0)	0.28^1^	0.48^1^
p-ADL (BADL)^i^, median (IQR)	17.0 (12 to 19)	17.0 (13.8 to 19)	18.0 (11 to 20)	0.71^1^	0.35^1^
i-ADL (NEADL)^j^, median (IQR)	26.0 (9 to 48)	31.0 (11.3 to 48)	31.0 (9 to 52)	0.50^1^	0.92^1^
CDR^k^, sum of boxes, median (IQR)	2.0 (0 to 9)	2.8 (0 to 10)	1.0 (0 to 8)	0.54^1^	0.14^1^
Depressive symptoms^l^, n (%)	102 (38.1)	114 (41.0)	59 (47.6)	0.48^2^	0.58^2^

*IQR* Inter quartile range. *SPPB* Short Physical Performance Battery. *MMSE* Mini Mental State Evaluation. *p-ADL* Personal Activities of Daily Living. *BADL* Barthel Activities of Daily Living Index. *i-ADL* Instrumental Activities of Daily Living. *NEADL* Nottingham Extended Activities of Daily Living. *CDR* Clinical Dementia Rating Scale

A. Extracapsular fracture vs. Intracapsular fracture (hemiarthroplasty)

B. Intracapuslar fracture (hemiarthroplasty) vs. intracapsular fracture (internal fixation)

^a^ Measured as hours from admission to start of anesthesia. Missing in 2 patients with an intracapsular fracture operated with hemiarthroplasty

^b^ Measured as duration of anesthesia. Missing in 8 patients with an extracapsular fracture, 10 patients with an intracapsular fracture operated with hemiarthroplasty, and 3 patients with an intracapsular fracture operated with internal fixation

^c^ Missing in 5 patients with an extracapsular fracture, 4 patients with an intracapsular fracture operated with hemiarthroplasty, and 3 patients with an intracapsular fracture operated with internal fixation

^d^ Missing in 1 patient with an extracapsular fracture and 1 patient with an intracapsular fracture operated with hemiarthroplasty 4-months follow-up. Missing in 1 patient with an intracapsular fracture operated with internal fixation at 12-months follow-up

^e^ Missing in 34 patients with an extracapsular fracture, 32 patients with an intracapsular fracture operated with hemiarthroplasty, and 22 patients with an intracapsular fracture operated with internal fixation at 4-months follow-up. Missing in 35 patients with an extracapsular fracture, 42 patients with an intracapsular fracture operated with hemiarthroplasty, and 17 patients with an intracapsular fracture operated with internal fixation at 12-months follow-up

^f^ Missing in 9 patients with an extracapsular fracture, 11 patients with an intracapsular fracture operated with hemiarthroplasty, and 8 patients with an intracapsular fracture operated with internal fixation at 4-months follow-up. Missing in 15 patients with an extracapsular fracture, 10 patients with an intracapsular fracture operated with hemiarthroplasty, and 6 patients with an intracapsular fracture operated with internal fixation at 12-months follow-up

^g^ Missing in 41 patients with an extracapsular fracture, 31 patients with an intracapsular fracture operated with hemiarthroplasty, and 22 patients with an intracapsular fracture operated with internal fixation at 4-months follow-up. Missing in 35 patients with an extracapsular fracture, 31 patients with an intracapsular fracture operated with hemiarthroplasty, and 20 patients with an intracapsular fracture operated with internal fixation at 12-months follow-up

^h^ Missing in 15 patients with an extracapsular fracture, 20 patients with an intracapsular fracture operated with hemiarthroplasty, and 15 patients with an intracapsular fracture operated with internal fixation at 4-months follow-up. Missing in 15 patients with an extracapsular fracture, 16 patients with an intracapsular fracture operated with hemiarthroplasty, and 5 patients with an intracapsular fracture operated with internal fixation at 12-months follow-up

^i^ Missing in 5 patients with an extracapsular fracture, 5 patients with an intracapsular fracture operated with hemiarthroplasty, and 5 patients with an intracapsular fracture operated with internal fixation at 4-months follow-up. Missing in 7 patients with an extracapsular fracture, 11 patients with an intracapsular fracture operated with hemiarthroplasty, and 1 patient with an intracapsular fracture operated with internal fixation at 12-months follow-up

^j^ Missing in 12 patients with an extracapsular fracture, 7 patients with an intracapsular fracture operated with hemiarthroplasty, and 8 patients with an intracapsular fracture operated with internal fixation at 4-months follow-up. Missing in 5 patients with an extracapsular fracture, 11 patients with an intracapsular fracture operated with hemiarthroplasty, and 1 patient with an intracapsular fracture operated with internal fixation at 12-months follow-up

^k^ Missing in 15 patients with an extracapsular fracture, 16 patients with an intracapsular fracture operated with hemiarthroplasty, and 15 patients with an intracapsular fracture operated with internal fixation at 4-months follow-up. Missing in 12 patients with an extracapsular fracture, 11 patients with an intracapsular fracture operated with hemiarthroplasty, and 1 patient with an intracapsular fracture operated with internal fixation at 12-months follow-up

^l^ Missing in 10 patients with an extracapsular fracture, 17 patients with an intracapsular fracture operated with hemiarthroplasty, and 8 patients with an intracapsular fracture operated with internal fixation at 4-months follow-up. Missing in 16 patients with an extracapsular fracture, 18 patients with an intracapsular fracture operated with hemiarthroplasty, and 5 patients with an intracapsular fracture operated with internal fixation at 12-months follow-up

^1^ Mann-Whitney U Test

^2^ Chi-Square Test

At four months follow-up, patients with an intracapsular fracture had significantly better median SPPB scores (4.0 vs. 3.0, *p* = 0.014) and better median p-ADL scores (17.0 vs. 16.0, *p* = 0.014) (Table 4), which did not persist at 12 months follow-up. There were no other differences between the two groups at four- and 12-months follow-up, see Table 4.

### Intracapsular hip fracture operated with HA vs internal fixation

Of those with an intercapsular fracture, those operated with an internal fixation at admission had lower BMI (median (IQR) 22 [20, 21, 22, 23, 24, 25, 26] vs. 24 [22, 23, 24, 25, 26, 27], *p* < 0.001) and more comorbidities (CCI 2 [1, 2, 3, 4] vs. 2(0–3), *p* = 0.008. Table 2). There were no other significant differences at admission. The same group had shorter surgery time (107(89–141) vs. 179(156–200) minutes, *p* < 0.001) and also shorter LOS (8 [6, 7, 8, 9, 10, 11, 12] vs. 11 [8, 9, 10, 11, 12, 13, 14] days, *p* < 0.001, Table 4 and supplementary Table 2. Fewer died during the hospital stay. At the four month control there were no differences between groups. The only difference at the 12 months control was slightly better gait speed in the group with internal fixation.

Sensitivity analyses including home-dwelling patients aged 70 years or older showed that there was no difference between the groups regarding waiting time for surgery, LOS or mortality rate during hospital stay, see Supplementary Table 3. There were no other differences between the groups at either four- or 12-months follow-up (Supplementary Table 3).

## Discussion

In the present study, including 711 hip fracture patients, we found no difference in mortality rate, new nursing home admissions or LOS between the patients with an extracapsular and an intracapsular fracture. Patients with an intracapsular fracture had better mobility, p-ADL and i-ADL at four months follow-up, but the differences did not persist at 12 months follow-up.

Sub-group analysis comparing extracapsular fracture patients with intracapsular fracture patients operated with HA, showed similar results with better mobility and p-ADL at four months follow-up, which did not persist 1 year postoperatively.

Previous studies comparing intracapsular and extracapsular hip fractures report worse outcomes for the patients having an extracapsular fracture [32, 53, 54]. Kristensen et al. conducted an observational study on 280 hip fracture patients in Denmark, finding that patients with an extracapsular fracture were four times more likely to not regain their independence in basic mobility, as well as having longer LOS and being less likely to be discharged to their own home, compared to patients with an intracapsular fracture [53]. Karagiannis et al. followed 499 hip fracture patients for 10 years, and found that the mortality rate at 5 and 10 years after hip fracture were higher for the patients suffering an extracapsular fracture, but the mortality rate was similar 1 year after surgery [54]. Our findings differ from common perception in the literature. We found no differences between the two groups in regards to LOS, mortality rate and new nursing home admissions, neither when comparing extracapsular fractures with all intracapsular fractures nor when limiting the intracapsular fracture group to those operated with HA. However, we only followed patients for 1 year, and thus cannot account for the changes and/or differences in mortality rates between the groups later than that. In addition, patients admitted from a nursing home are underrepresented in our sample since they were excluded in the Trondheim Trial. These frailer patients would reasonably have contributed to a higher mortality rate and longer LOS.

We did, however, find a difference in physical function measured by SPPB and p-ADL measured by BADL in favor of the intracapsular fracture group at four months, when comparing the extracapsular fracture and intracapsular fracture (operated with HA) groups. This difference did not persist after 1 year (Table 4). As a difference of 0.5 points in SPPB [55] and one point in BADL is roughly equivalent with being able to go to the toilet or not, these differences were deemed clinically relevant. Cut-point estimates for clinical relevance, like MCID or MCII, varies with patient characteristics, type of intervention undergone, estimation technique and time to follow-up [56]. A prospective cohort study including 170 women with a hip fracture had similar results – the patients with an extracapsular fracture had worse function at discharge, but the difference did not persist after one year [27]. This may be due to different rehabilitation needs in the two sub-groups of hip fracture patients, and might reflect that patients with an intracapsular fracture operated with HA have a faster initial recovery, but that both groups reach the same functional level later. The advantage of intracapsular fractures treated with HA, is the removal of the fracture completely, in contrast to extracapsular fractures treated with osteosynthesis, which then needs more time to heal. In addition, more hidden blood loss, larger edema and more pain in extracapsular hip fractures might partly explain the initial differences [33]. Thus, patients with an extracapsular fracture have less ability to bear weight on the affected limb postoperatively, which can initially impede rehabilitation [35]. Patients with an extracapsular fracture may therefore need more time and/or intensified rehabilitation after the fracture to reach the same functional levels as patients with an intracapsular fracture [28, 32].

When excluding patients admitted from a nursing home, there were no longer statistically significant differences between the groups in SPPB or ADL measures. The explanation might be that the frailest patients admitted for a hip fracture were admitted from a nursing home. Thus, they might have less capacity for rehabilitation and therefore are the contributing factor to the difference between groups [39]. This is in line with previous literature finding that the youngest and more independent patients benefited most from a rehabilitation intervention [9]. However, it may also be due to a loss of statistical power after excluding patients. In our material, 56 nursing home patients had an intracapsular fracture and 45 nursing home patients had an extracapsular fracture.

A common perception is that patients with an intracapsular fracture differ from patients with an extracapsular fracture at the time of fracture, with better baseline characteristics [28]. In this study, the groups were similar with regard to age, sex, comorbidities, ADL function, cognitive function and physical function at baseline. However, the differences between groups might have been larger if a more representative number of nursing home patients had been included.

The care of hip fracture patients has improved over the last decades, with more emphasis on early rehabilitation, polypharmacy optimization, physiotherapy, and cooperation between orthopedic surgeons and geriatricians. This might have contributed to the reduced reported differences in outcome between the extracapsular and intracapsular hip fracture patients’ postoperative results and mortality [9].

Several methodological limitations should be taken into consideration when interpreting our results. One is that we performed post hoc exploratory analyses. Some outcomes collected at baseline were based on proxy interviews, and could therefore have been biased by the knowledge of the recent fracture. We only conducted bivariate analyses, and thus our results should be interpreted with caution as confounding factors might be present. This could include the intervention provided in the original RCTs these patients were recruited from (orthogeriatric care in both studies). Additional confounding factors include pre-trauma ADL, age, sex, and pre-trauma dementia. However, the groups were similar at baseline. Unfortunately, the study did not have information on specific types of surgical treatment for each subgroup. Strengths of this study include the high number of participants that are followed for one year after a hip fracture, and the systematic and comprehensive collection of outcomes. The study includes a large and heterogeneous sample of patients with a hip fracture, which are representative of older adults in the population, and thus might allow for generalizability of our findings.

## Conclusion

We found no differences in mortality rate, LOS or new nursing home admissions between patients suffering from an extracapsular versus an intracapsular hip fracture. Patients operated with a HA for an intracapsular fracture had better function measured by SPPB (mobility) and BADL (p-ADL) after 4 months, which did not persist 12 months after surgery. This may indicate a more rapid rehabilitation after HA. Although we did not find major differences between groups after 12 months, our study highlights the transient differences in postoperative recovery profiles between extracapsular and intracapsular hip fractures. The first months after the fracture is when extracapsular fractures experience diminished functional outcomes, and this suggests that early and intensive rehabilitation is particularly important in this group.

## Electronic supplementary material

Below is the link to the electronic supplementary material.

Supplementary Material

## Data Availability

Due to a statement by the Data Protection Officer at Oslo University Hospital, data cannot be shared publicly because it is confidential (due to the consent given by the participants when included in the study). It is possible to extract information, upon request. Proposals should be directed to the Regional Ethics Committee for medical research in the South-East of Norway (contact: post@helseforsikring.etikkom.no).

## Abbreviations

ASA: The American Society of Anesthesiologists
BADL: Barthel Activities of Daily Living Index
CCI: Charlson Comorbidity Index
CDR: Clinical Dementia Rating Scale
CSDD: Cornell Scale of Depression in Dementia
GSD: 15–Geriatric Depression Scale 15
HA: Hemiarthroplasty
i-ADL: instrumental Activitites of Daily Living
LOS: Length of hospital stay
MMSE: Mini Mental Status EvaluationNEADL–Nottingham Extended Activities of Daily Living
p-ADL: Personal Activities of Daily Living
SPPB: Short Physical Performance battery
