# Supplementary material for: Postoperative outcomes in patients operated for extra- and intracapsular hip fractures – a secondary analysis of two randomized controlled trials
Source: BMC Musculoskelet Disord. 2025 Feb 22;26:182. doi: 10.1186/s12891-025-08404-6 (PMC11846164; doi:10.1186/s12891-025-08404-6)
Supplement: Supplementary file 1 — Supplementary Material [file 12891_2025_8404_MOESM1_ESM.docx]

**Supplementary Table 1.**

Patient characteristics for the sensitivity analysis excluding patients admitted from a nursing home, and patients <70 years old.

|  | **Extracapsular  fracture**  **N=224** | **Intracapsular  fracture (hemiarthroplasty)**  **N=243** | **Intracapsular fracture (internal fixation) N=105** |
| --- | --- | --- | --- |
| Age (years), median (range) | 84.0 (70 to 97) | 85.0 (70 to 101) | 84.0 (70 to 99) |
| Gender, female, n (%) | 173 (77.2) | 188 (77.4) | 75 (71.4) |
| BMI^a^, median (IQR) | 23.9 (21.2 to 27.1) | 24.3 (21.4 to 26.5) | 21.7 (19.6 to 25.6) |
| ASA score^b^, median (IQR) | 3.0 (2 to 3) | 3.0 (2 to 3) | 3.0 (2 to 3) |
| CCI, median (IQR) | 2.0 (0 to 3) | 2.0 (1 to 3) | 2.0 (1 to 4) |
| Grip strength^c^ (kg), median (IQR) | 18.0 (14 to 25) | 20.0 (15 to 26) | 20.0 (14.8 to 28) |
| p-ADL (BADL)^d^, median (IQR) | 19.0 (17 to 20) | 19.0 (17 to 20) | 20.0 (17 to 20) |
| i-ADL (NEADL)^e^, median (IQR) | 44.5 (28 to 56) | 42.0 (25 to 57) | 46.0 (29 to 58.8) |
| CDR^f^, sum of boxes, median (IQR) | 1.0 (0 to 4) | 1.0 (0 to 5.4) | 1.0 (0 to 3) |

*IQR* Inter quartile range. *BMI* Body Mass Index. *ASA* American Society of Anesthesiologists. *CCI* Charlson Comorbidity Index. *P-ADL* Personal Activities of Daily Living. *BADL* Barthel Activities of Daily Living Index. *I-ADL* Instrumental Activities of Daily Living. *NEADL* Nottingham Extended Activities of Daily Living. *CDR* Clinical Dementia Rating Scale.

^a^ Missing in 97 patients with an extracapsular fracture, 103 patients with an intracapsular fracture operated with hemiarthroplasty, and 48 patients with an intracapsular fracture operated with internal fixation.

^b^ Missing in 6 patients with an extracapsular fracture, 3 patients with an intracapsular fracture operated with hemiarthroplasty, and 4 patients with an intracapsular fracture operated with internal fixation.

^c^ Missing in 21 patients with an extracapsular fracture, 28 patients with an intracapsular fracture operated with hemiarthroplasty, and 11 patients with an intracapsular fracture operated with internal fixation.

^d^ Missing in 4 patients with an extracapsular fracture, 5 patients with an intracapsular fracture operated with hemiarthroplasty, and 1 patient with an intracapsular fracture operated with internal fixation.

^e^ Missing in 6 patients with an extracapsular fracture, 8 patients with an intracapsular fracture operated with hemiarthroplasty, and 1 patient with an intracapsular fracture operated with internal fixation.

^f^ Missing in 18 patients with an extracapsular fracture, 15 patients with an intracapsular fracture operated with hemiarthroplasty, and 8 patients with an intracapsular fracture operated with internal fixation.

**Supplementary Table 2.**

Kaplan-Meier survival analysis and Log-Rank tests conducted to compare time-based outcomes between different fracture types and surgical interventions. Comparisons include the length of hospital stay (LOS) and duration of surgery across extracapsular versus intracapsular fractures, as well as intracapsular hemiarthroplasty (HA) versus intracapsular internal fixation (osteosynthesis). Results are stratified to exclude patients under the age of 70, as noted. The p-values indicate the statistical significance of differences between groups.

| **Analysis Type** | **Comparison Groups** | **Conclusion** | **p-value** |
| --- | --- | --- | --- |
| **Length of Stay (LOS)** | Extracapsular vs Intracapsular | No significant difference in LOS | 0.19 |
|  | Extracapsular vs Intracapsular HA (excluding patients under 70 years) | No significant difference in LOS | 0.86 |
|  | Intracapsular HA vs Intracapsular Osteosynthesis (excluding patients under 70 years) | LOS longer in Intracapsular HA | 0.003 |
| **Duration of Surgery** | Extracapsular vs Intracapsular | Duration longer in Intracapsular | <0.001 |
|  | Extracapsular vs Intracapsular HA (excluding patients under 70 years) | Duration longer in Intracapsular HA | <0.001 |
|  | Intracapsular HA vs Intracapsular Osteosynthesis (excluding patients under 70 years) | Duration longer in Intracapsular HA | <0.001 |

HA: Hemiarthroplasty. LOS: Length of stay

**Supplementary Table 3.**

Sensitivity analysis excluding patients admitted from a nursing home, and patients < 70 years

old. Outcomes at hospital stay, 4- and 12 months follow-up.

|  | **Extracapsular  fracture** | **Intracapsular  fracture (hemiarthroplasty)** | **Intracapsular fracture (internal fixation)** | **p-value**  **(A)** | **p-value (B)** |
| --- | --- | --- | --- | --- | --- |
| **Hospital outcomes** | | | | | |
| **N** | **224** | **243** | **105** |  |  |
| Hours to surgery^a^, median (IQR) | 22.5 (15 to 37) | 25.0 (17.8 to 41.8) | 23.0 (14.5 to 40.8) | 0.06^1^ | 0.19^1^ |
| Duration of operation^b^ (min), median (IQR) | 145 (125 to 175) | 180.0 (155 to 205) | 100.0 (90 to 129) | <0.001^1^ | <0.001^1^ |
| Type of anesthesia^c^, local/regional, n (%) | 210 (93.8) | 230 (94.7) | 99 (94.3) | 0.68^2^ | 0.71^2^ |
| Type of operation  Hemiarthroplasty, n (%)  Internal fixation, n (%) | 3 (1.3)  221 (97.8) | 243 (100) - | - 105 (100) | - | - |
| Length of hospital stay in days, median (IQR) | 11.0 (8 to 15) | 11.0 (8 to 14) | 9.0 (7 to 12) | 0.37^1^ | 0.003^1^ |
| Died during hospital stay, n (%) | 2 (0.9) | 6 (2.5) | 0 (0) | 0.19^2^ | 0.10^2^ |
| **4-months follow up** | | | | | |
| **N** | **180** | **199** | **98** |  |  |
| Living in a nursing home^d^, n (%) | 37 (16.5) | 38 (15.6) | 22 (21.0) | 0.79^2^ | 0.45^2^ |
| Died before 4-month follow-up, n (%) | 25 (11.2) | 24 (9.9) | 6 (5.7) | 0.65^2^ | 0.20^2^ |
| Grip strength^e^ (kg), median (IQR) | 18.0 (14 to 23.8) | 19.0 (14 to 25) | 20.0 (14 to 23.8) | 0.15^1^ | 0.61^1^ |
| SPPB^f^, median (IQR) | 4.0 (2 to 6.8) | 5.0 (2 to 7) | 6.0 (2 to 9) | 0.11^1^ | 0.33^1^ |
| Gait speed^g^ (m/s), median (IQR) | 0.54 (0.4 to 0.7) | 0.56 (0.4 to 0.7) | 0.61 (0.5 to 0.8) | 0.41^1^ | 0.20^1^ |
| MMSE^h^, median (IQR) | 25.0 (21 to 28) | 25.0 (21.2 to 28) | 25.0 (21 to 28) | 0.81^1^ | 0.92^1^ |
| p-ADL (BADL)^i^, median (IQR) | 17.0 (13 to 19) | 17.0 (15 to 20) | 18.0 (13 to 20) | 0.17^1^ | 0.54^1^ |
| i-ADL (NEADL)^j^, median (IQR) | 32.0 (11 to 46) | 34.5 (13 to 49) | 36.0 (13 to 52) | 0.42^1^ | 0.67^1^ |
| CDR^k^, sum of boxes, median (IQR) | 1.0 (0 to 6) | 1.0 (0 to 7) | 0.0 (0 to 5) | 0.57^1^ | 0.24^1^ |
| Depressive symptoms^l^, n (%) | 115 (51.3) | 115 (47.3) | 60 (57.1) | 0.57^2^ | 0.70^2^ |
| **12-months follow-up** | | | | | |
| **N** | **159** | **177** | **83** |  |  |
| Living in a nursing home^d^, n (%) | 34 (15.2) | 44 (18.1) | 21 (20.0) | 0.39^2^ | 0.85^2^ |
| Died before 12-month follow-up, n (%) | 41 (18.3) | 39 (16.0) | 15 (14.3) | 0.52^2^ | 0.68^2^ |
| Grip strength^e^ (kg), median (IQR) | 18.0 (14 to 24) | 19.0 (14 to 24.8) | 18.0 (14 to 25) | 0.46^1^ | 0.75^1^ |
| SPPB^f^, median (IQR) | 4.0 (2 to 7) | 4.0 (2 to 8) | 5.0 (2 to 8) | 0.69^1^ | 0.56^1^ |
| Gait speed^g^ (m/s), median (IQR) | 0.56 (0.4 to 0.8) | 0.57 (0.4 to 0.8) | 0.64 (0.5 to 0.9) | 0.62^1^ | 0.021^1^ |
| MMSE^h^, median (IQR) | 25.5 (21 to 28) | 24.0 (19 to 27) | 25.0 (20.8 to 27) | 0.08^1^ | 0.29^1^ |
| p-ADL (BADL)^i^, median (IQR) | 18.0 (14.9 to 20) | 17.0 (15 to 20) | 19.0 (13.5 to 20) | 0.72^1^ | 0.17^1^ |
| i-ADL (NEADL)^j^, median (IQR) | 34.0 (15 to 49) | 34.0 (12 to 51) | 35.5 (12 to 53.3) | 0.98^1^ | 0.61^1^ |
| CDR^k^, sum of boxes, median (IQR) | 1.0 (0 to 6) | 2.0 (0 to 8) | 0.0 (0 to 5.3) | 0.11^1^ | 0.046^1^ |
| Depressive symptoms^l^, n (%) | 98 (43.8) | 110 (45.3) | 58 (55.2) | 0.88^2^ | 0.38^2^ |

*IQR* Inter quartile range. *SPPB* Short Physical Performance Battery. *MMSE* Mini Mental State Evaluation. *p-ADL* Personal Activities of Daily Living. *BADL* Barthel Activities of Daily Living Index. *i-ADL* Instrumental Activities of Daily Living. *NEADL* Nottingham Extended Activities of Daily Living. *CDR* Clinical Dementia Rating Scale.

A. Extracapsular fracture vs. Intracapsular fracture (hemiarthroplasty)

B. Intracapuslar fracture (hemiarthroplasty) vs. intracapsular fracture (internal fixation)

^a^ Measured as hours from admission to start of anesthesia. Missing in 2 patients with an intracapsular fracture operated with hemiarthroplasty.

^b^ Measured as duration of anesthesia. Missing in 5 patients with an extracapsular fracture, 8 patients with an intracapsular fracture operated with hemiarthroplasty, and 2 patients with an intracapsular fracture operated with internal fixation.

^c^ Missing in 4 patients with an extracapsular fracture, 4 patients with an intracapsular fracture operated with hemiarthroplasty, and 3 patients with an intracapsular fracture operated with internal fixation.

^d^ Missing in 1 patient with an extracapsular fracture and 1 patient with an intracapsular fracture operated with hemiarthroplasty at 4-months follow-up. Missing in 1 patient with an intracapsular fracture operated with internal fixation at 12-months follow-up.

^e^ Missing in 24 patients with an extracapsular fracture, 24 patients with an intracapsular fracture operated with hemiarthroplasty, and 14 patients with an intracapsular fracture operated with internal fixation at 4-months follow-up. Missing in 29 patients with an extracapsular fracture, 37 patients with an intracapsular fracture operated with hemiarthroplasty, and 12 patients with an intracapsular fracture operated with internal fixation at 12-months follow-up.

^f^ Missing in 8 patients with an extracapsular fracture, 8 patients with an intracapsular fracture operated with hemiarthroplasty, and 7 patients with an intracapsular fracture operated with internal fixation at 4-months follow-up. Missing in 11 patients with an extracapsular fracture, 10 patients with an intracapsular fracture operated with hemiarthroplasty, and 4 patients with an intracapsular fracture operated with internal fixation at 12-months follow-up.

^g^ Missing in 25 patients with an extracapsular fracture, 20 patients with an intracapsular fracture operated with hemiarthroplasty, and 15 patients with an intracapsular fracture operated with internal fixation at 4-months follow-up. Missing in 20 patients with an extracapsular fracture, 24 patients with an intracapsular fracture operated with hemiarthroplasty, and 14 patients with an intracapsular fracture operated with internal fixation at 12-months follow-up.

^h^ Missing in 10 patients with an extracapsular fracture, 19 patients with an intracapsular fracture operated with hemiarthroplasty, and 11 patients with an intracapsular fracture operated with internal fixation at 4-months follow-up. Missing in 12 patients with an extracapsular fracture, 15 patients with an intracapsular fracture operated with hemiarthroplasty, and 5 patients with an intracapsular fracture operated with internal fixation at 12-months follow-up.

^i^ Missing in 4 patients with an extracapsular fracture, 5 patients with an intracapsular fracture operated with hemiarthroplasty, and 5 patients with an intracapsular fracture operated with internal fixation at 4-months follow-up. Missing in 7 patients with an extracapsular fracture, 10 patients with an intracapsular fracture operated with hemiarthroplasty, and 1 patient with an intracapsular fracture operated with internal fixation at 12-months follow-up.

^j^ Missing in 9 patients with an extracapsular fracture, 7 patients with an intracapsular fracture operated with hemiarthroplasty, and 7 patients with an intracapsular fracture operated with internal fixation at 4-months follow-up. Missing in 5 patients with an extracapsular fracture, 10 patients with an intracapsular fracture operated with hemiarthroplasty, and 1 patient with an intracapsular fracture operated with internal fixation at 12-months follow-up.

^k^ Missing in 15 patients with an extracapsular fracture, 16 patients with an intracapsular fracture operated with hemiarthroplasty, and 15 patients with an intracapsular fracture operated with internal fixation at 4-months follow-up. Missing in 13 patients with an extracapsular fracture, 11 patients with an intracapsular fracture operated with hemiarthroplasty, and 1 patient with an intracapsular fracture operated with internal fixation at 12-months follow-up.

^l^ Missing in 6 patients with an extracapsular fracture, 16 patients with an intracapsular fracture operated with hemiarthroplasty, and 6 patients with an intracapsular fracture operated with internal fixation at 4-months follow-up. Missing in 14 patients with an extracapsular fracture, 15 patients with an intracapsular fracture operated with hemiarthroplasty, and 4 patients with an intracapsular fracture operated with internal fixation at 12-months follow-up.

^1^ Mann-Whitney U Test

^2^ Chi-Square Test
